# A supervised land cover classification of a western Kenya lowland endemic for human malaria: associations of land cover with larval *Anopheles *habitats

**DOI:** 10.1186/1476-072X-8-19

**Published:** 2009-04-16

**Authors:** FM Mutuku, MN Bayoh, AW Hightower, JM Vulule, JE Gimnig, JM Mueke, FA Amimo, ED Walker

**Affiliations:** 1 Centers for Disease Control and Prevention/Kenya Medical Research Institute, Kisumu, Kenya; 2 Centre for Global Health Research, Kenya Medical Research Institute, Kisumu, Kenya; 3 Division of Parasitic Diseases, Centers for Disease Control and Prevention, Atlanta, GA, USA; 4 Department of Zoological Sciences, Kenyatta University, Nairobi, Kenya; 5 Department of Biology, University of Eastern Africa, Baraton, Kenya; 6 Department of Microbiology and Molecular Genetics, Michigan State University, East Lansing, MI, USA

## Abstract

**Background:**

A supervised land cover classification was developed from very high resolution IKONOS satellite data and extensive ground truth sampling of a ca. 10 sq km malaria-endemic lowland in western Kenya. The classification was then applied to an investigation of distribution of larval *Anopheles *habitats. The hypothesis was that the distribution and abundance of aquatic habitats of larvae of various species of mosquitoes in the genus *Anopheles *is associated with identifiable landscape features.

**Results and discussion:**

The classification resulted in 7 distinguishable land cover types, each with a distinguishable vegetation pattern, was highly accurate (89%, Kappa statistic = 0.86), and had a low rate of omission and commission errors. A total of 1,198 habitats and 19,776 *Anopheles *larvae of 9 species were quantified in samples from a rainy season, and 184 habitats and 582 larvae from a dry season. *Anopheles gambiae *s.l. was the dominant species complex (51% of total) and *A. arabiensis *the dominant species. Agricultural land covers (mature maize fields, newly cultivated fields, and pastured grasslands) were positively associated with presence of larval habitats, and were located relatively close to stream channels; whilst nonagricultural land covers (short shrubs, medium shrubs, tall shrubs, and bare soil around residences) were negatively associated with presence of larval habitats and were more distant from stream channels. Number of larval habitats declined exponentially with distance from streams. IKONOS imagery was not useful in direct detection of larval habitats because they were small and turbid (resembling bare soil), but was useful in localization of them through statistical associations with specific land covers.

**Conclusion:**

A supervised classification of land cover types in rural, lowland, western Kenya revealed a largely human-modified and fragmented landscape consisting of agricultural and domestic land uses. Within it, larval habitats of *Anopheles *vectors of human malaria were associated with certain land cover types, of largely agricultural origin, and close to streams. Knowledge of these associations can inform malaria control to gather information on potential larval habitats more efficiently than by field survey and can do so over large areas.

## Background

Malaria transmission is dependent upon presence of populations of susceptible *Anopheles *mosquitoes which feed upon man. The distribution and abundance of these vector mosquitoes is strongly associated with landscape features such as topography, vegetation, and soil [1-3], owing to the particular habitat requirements of the aquatic, larval stages [4-6]. For example, Hightower *et al*. [7] observed positive associations between distribution of adult mosquitoes, prevalence of malaria parasitemia in humans, and distance of human residence to larval habitats in a rural region of Nyanza Province, western Kenya. The distribution of the most efficient malaria vector species in sub-Saharan Africa, *Anopheles gambiae*, is influenced by particular topographic and environmental factors which in turn influence the location and productivity of the larval habitats in both lowland and highland [8-10] regions of western Kenya, where malaria is highly endemic [7,11].

Land cover is one landscape feature that likely plays a central role in epidemiology of malaria. Conversion of natural papyrus marshes to drained fields for cultivation of crops resulted in increased local temperature, creation of suitable *A. gambiae *s.l. larval habitats, and elevated risk of epidemic malaria transmission in a highland region of southwestern Uganda [12]. In highlands of western Kenya, Minakawa et al. [13] found significantly more *A. gambiae *s.l. larval habitats located in farmlands along valley bottoms, compared to nearby forests, swamps, road ways, and pastures in both wet and dry seasons. Higher water temperatures associated with the habitats located in farmlands enhanced development of the aquatic stages of *A. gambiae *[14]. In the same study, occurrence of *A. gambiae *larvae was negatively associated with canopy cover and emergent plants in natural habitats located in forest and swamp land cover types. These findings suggest that variation in landscape structure is important to bionomics of malaria vectors and to malaria transmission and that such variations may be related to land use, vegetation, and microclimate. However, the influence of landscape structure in the context of a comprehensive, supervised classified land cover on these relationships has not been quantified in a lowland malaria endemic setting.

The application of remote sensing (RS) technology and geographical information systems (GIS) in malaria epidemiology has increased greatly in recent years. Initial studies employed low to high spatial resolution satellite data [15-18]. Low spatial resolution was useful for general studies on broad zones and regions [1,19,20]. However, new very high resolution instruments (such as IKONOS, 3.2 m spatial resolution and QUICKBIRD, 2.4 m spatial resolution) are being utilized in different ways in research on malaria vectors, such as in production of digitized land cover maps [13] and in attempts to locate directly larval habitats [10]. Ground-based surveys and studies focused on characterizing *A. gambiae *larval habitats biologically, chemically and physically have been conducted in western Kenya [13,21-23]. However, the specific land cover types that are likely to harbor larval habitats have not been extensively explored.

The goal of the present study was several fold. Primarily, it was to develop a supervised classification of land cover in a rural area of lowland, western Kenya towards associating identified land cover types with distribution of larval habitats of *Anopheles *mosquitoes. Secondarily, the goal was to determine whether very high spatial resolution imagery (IKONOS) provided sufficient resolution to identify larval habitats directly.

## Methods

### Study site

The study site has been the focus of intensive malariological studies and is described elsewhere [24]. The area under study encompassed part of the communities of Asembo and Seme, was 3.216 × 3.216 km (10.34 km^2^) in area, and was located at 34°23'E, 0°11'S in Nyanza Province, western Kenya, approximately 50 km west of the city of Kisumu. Physiographically, the site falls within the Yala-Nzoia Plains, a lake lowlands region overlying granite bedrock and surrounded by Nyanza Low Plateau topography, and bordering the north shore of Winam Gulf of Lake Victoria [25]. Most human inhabitants are subsistence farmers who cultivate maize, cassava, and vegetables; and husband cattle, goats, sheep, and chickens. Fishing in nearby Lake Victoria is an important local economic activity. Rainfall occurs year-round with two main peaks; the long rains falling between March and May, and the short rains between November and December [26]. Total annual rainfall averages 1400 mm per year and daily temperatures range from 25.5°C to 33.0°C [24]. Malaria due to infection with *Plasmodium falciparum *is holoendemic in the region with transmission occurring throughout the year. The principal malaria vectors in the area are two species in the *Anopheles gambiae *sensu latu complex (namely, *A. gambiae *sensu strictu and *A. arabiensis*), and *A. funestus *[24,27].

Studies on the distribution, location and productivity of *A. gambiae *larval habitats in a hilly, highland region of western Kenya have demonstrated that productive habitats are located in valley bottoms near streams and with agricultural crops being the predominant land cover type [10,13,14]. By contrast, our study area is lowland plain with some gentle, rolling hills, and with temporary streams that become inundated during the rainy season. These streams and numerous, small, man-made water reservoirs serve as water sources in the dry season, as does the lake. Clusters of houses (i.e., compounds), streams, roads and other physical features have been mapped previously [7,24].

### IKONOS satellite image

An IKONOS satellite image (Space Imaging, Atlanta, Georgia) of the study area was acquired to primarily identify land cover types associated with location of larval habitats for *Anopheles *mosquitoes. The IKONOS scene acquired was 100 km^2^, of which 10.34 km^2 ^were used in this study. The image was acquired on May 17, 2005 at 8.14 GMT, and was centered on 34.435571E and 0.143949S. The image was registered and orthorectified with geographic latitude-longitude coordinates using ground reference points taken with a global positioning system (GPS). Because changes in land cover types in the study area are mainly due to seasonal cropping practices and the deciduous nature of most plant species, only one IKONOS image was used for both dry and wet seasons.

### Larval habitat surveys

Two surveys for aquatic larval habitats were conducted in the study area from May to June for the wet season, and in October for the dry season. During each survey, all pools of standing and relatively slow moving water were quantitatively sampled using an area sampling method as described by Mutuku *et al*. [9]. The term "potential habitat" in this study refers to any discrete body of water that is likely to remain inundated for at least 2 days. These habitats were categorized into seven types including drainage channels, burrow pits, rain pools, cluster of hoof prints, stream bed pools, wet meadows, and tire tracks [8]. In the dry season survey, the small temporary pools in the form of rain pools, drainage channels, cluster of hoof prints and wet meadows were absent.

### Ground sampling

To determine the accuracy of the land cover/use classification, the study area (measured 3.216 × 3.216 km covering an area of 10.34 km^2^) was divided into 36 grid cells of 500 m × 500 m each. With the assumption that distribution of different types of land cover is homogeneous in the study area, 16 of these grid cells were randomly selected for ground-truthing of land cover types. One individual, familiar with the territory, traversed each cell and at each of 951 locations mapped the different land cover types. The individual identified the appropriate land cover type to category by visual inspection, and completed a GPS reading of the center location of the land cover. This procedure was repeated for each neighboring land cover. The process took two weeks and was timed to coincide with the date when the IKONOS satellite image was acquired. These points were located using differential GPS with a precision of about 1 m and were used as ground reference points for comparison with remotely sensed data and the supervised classification. The number of points mapped in each of the 16 grid cells was proportional to the land cover heterogeneity in each particular grid cell.

### Supervised classification

The IKONOS imagery obtained from Space Imaging during the wet season was barely obscured by clouds and shadows, representing only 4% of the image area, leaving all of the study area where larval sampling occurred free of cloud cover. ERDAS IMAGINE 8.5 image processing software [28] was used for image processing and analysis. Through resolution merging, a pseudo-1- meter resolution image using the four multispectral bands and the panchromatic band was constructed using principal component analysis (PCA) in ERDAS IMAGINE 8.5 [28,29]. This fused image (henceforth referred to as the pan-sharpened IKONOS image) was visually more interpretable than either the 4-meter multispectral image or the 1-meter panchromatic image. The IKONOS image was classified through a combination of visual, unsupervised, and supervised methods. The ISODATA (Iterative Self-Organizing Data Analysis Technique) unsupervised classification algorithm was performed to differentiate the spectral clusters corresponding to the basic land cover types. The unsupervised classification used the green, red, and near-infrared spectral bands to produce 30 spectrally distinct classes, each based on the analyst's prior knowledge of the different land cover types in the study region. With the aid of the ground-truthing reference points, the unsupervised classified image, and the pan-sharpened IKONOS image, the multispectral IKONOS image was then visually classified according to typical land cover types and vegetation. This process resulted in 27 training signatures with most of them being spectrally close. The 27 training signatures (subclasses) were amalgamated to ten major training signatures (classes) including grasslands, water, mature maize, newly cultivated fields, clouds, shadows, bare land, short shrubs, medium shrubs, and tall shrubs (including trees). The subclasses helped define each class more accurately, for example, shallow water and deep water were used for water, 3 maize fields (subclasses) were trained for mature maize and 5 patches of grassland were used to train for grassland. The supervised classification was done on the entire IKONOS scene. Supervised classification was then performed, using a maximum likelihood classifier.

### Data analysis

To ensure that the information derived from the classification was of high quality and to deduce meaningful indications on thematic correctness [30], the classified image was assessed for accuracy using Cohen's Kappa and classification table metrics 2.C, an ArcView 3.3 extension (ESRI, Redlands, California). In the assessment, the classified IKONOS image and the ground reference data were compared. The relationship between these sets of information was summarized in two ways: (1) a confusion matrix, which describes the comparison of the remote sensing derived classification map and the ground truth reference data; and (2) the Kappa statistic, which provides a measure of agreement between the classified remotely sensed data and the ground reference data.

There were no ground reference data for the land cover classes termed "shadows", "clouds" and "water" in the classified image and hence these were assigned to a "No Data" classification. Newly cultivated fields were amalgamated with mature maize because most of the fields in the newly cultivated fields land cover class were actually maize fields. Overall accuracy and class-specific user and producer accuracies were calculated for each of the resultant six land cover classes. Producer's accuracy was obtained by taking the number of points classified correctly for a class divided by the number of ground reference points in that class, while the user's accuracy was the number of points classified correctly for a class divided by the number of points classified as that class [31]. When a point was incorrectly included in a class, an error of commission has occurred. Inversely, when a point was excluded from the proper class, an error of omission has occurred.

The spatial distribution of the larval habitats was created as a layer and overlaid on the land cover layer, and the number of larval habitats in each land cover class was calculated using Zonal statistics, a tool from the Spatial Analyst extension in ArcView GIS (ESRI, Redlands, California). Accuracy in identifying the land cover class where larval habitats were located was ensured by creating a 4 meter radius buffer around habitat so that larval habitats located at the edges land cover types will associated with land cover closet to them, in which the majority land cover type was identified. A range of buffer areas (4, 8, 12, 16, and 20 meters) was examined but the 4 meter radius buffer was supported well by the majority neighborhood statistic. Chi-square analysis for categorical variables was used to examine whether there were significant differences in proportions of positive and negative larval habitats located in different land cover classes. Tukey style multiple comparison of proportions were used for post-hoc analyses [32]. Seven categories of distance from streams were created using ArcGIS 9.1 (ESRI, Redlands, California), i.e., 100, 200, 300, 400, 500, 600, and >600 meter distance to the nearest stream centerline. These distance categories were overlaid on top of thematic maps of the classified IKONOS image to extract the proportions of each land cover type within the various distance categories. Regression analysis was used to determine the relationship between the proportions of the various land cover classes and the distance categories from stream channels.

### Results Habitat enumeration and larval abundance

**Figure 1:**
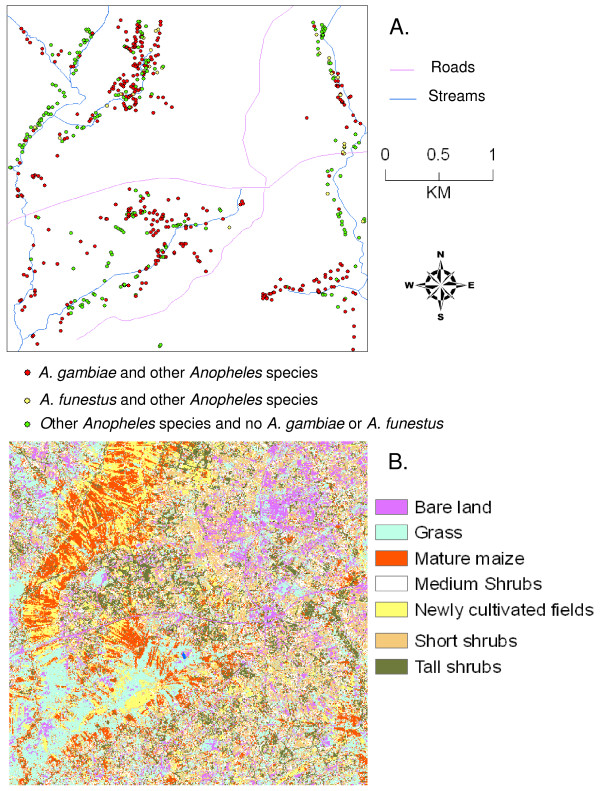
**A. Distribution of 1,198 larval *Anopheles *habitats in a rural lowland landscape of a western Kenya lake plain during the wet season**. B. Supervised classification of land cover in a rural lowland landscape in western Kenya lake plain, showing the distribution of seven classified land cover classes.

The distribution of habitats in the study area is shown in Figure 1A. In the wet season survey, 86% of the 1,198 potential habitats located and sampled had *Anopheles *larvae; in total 19,776 larvae were collected. In the dry season, 40% of the 184 potential habitats located and sampled were positive for *Anopheles *larvae and in total 582 larvae were collected. Of these larvae, 2,231 were mature and could be identified morphologically to species. Overall, *Anopheles gambiae *s.l. (a species complex of two important malaria vector species, *A. gambiae *s.s. and *A. arabiensis*) represented 50.69% of total, *A. coustani *was 21.07%, *A. rufipes *was 10.89%, *A. pharoensis *(5.15%), *A. squamosus *(4.71%), *A. maculipalpis *(4.71%), *A. funestus *(1.97%), *A. gibbensi *(0.40%), and *A. pretoriensis *(0.40%). Analysis of 1,078 of the larvae identified morphologically as *A. gambiae *s.l. by the PCR method [22] revealed that 796 (73.8%) were *A. arabiensis*, 19.8% were *A. gambiae *s.s., and 69 (6.4%) did not react in the test used.

### Results of the supervised IKONOS image classification

Classification results (Figure 1B) revealed that the landscape was highly modified by human activities typical of rural subsistence farming. Most of the study area was covered by grassland (21%), medium shrubs (16%), short shrubs (15%), mature maize (14%) and tall shrubs (13%). Shadow (mainly from tall shrubs) represented a very small proportion of the total land cover (<1%). It was combined with the tall shrubs land cover class. Bare land (10%) and newly cultivated fields (11%) were the least common land cover types. Twenty-nine percent of *Anopheles *larvae came from habitats sampled from the mature maize land cover type, whilst 26% were from grasslands, 17% from newly cultivated fields, and 13% from medium shrubs. Only 7% were sampled from the short shrubs land cover type, 6% from tall shrubs, and 3% from habitats located in the bare land type of land cover.

### Descriptions and uses of the land cover types

The vegetative community that dominated the **Grassland **land cover was primarily seasonal grasses, often grazed by cattle, and included *Hyparrhania ruffa *(Poaceae), *Cynodon dactlylon *(Poaceae), and *Eragrostis tenuifolia *(Poaceae). The **Bare Land **was a land cover in areas of exposed and typically dry soils, found along roads, near clusters of houses, and at school playgrounds. Corrugated iron roofs were captured by the supervised classification as being close to this land cover type, which is consistent with the bare soil around homesteads. **Mature Maize **was a dominant land cover, typified by numerous fields of maize (and occasionally sorghum) at a stage of mature growth. Maize plants in this class were generally about 2 to 3 meters tall, had tassels and were mainly found on low-lying areas along the streams. This class formed a closed canopy in most cases. In addition to crops grown in these fields, all the other vegetative communities mentioned under newly cultivated fields were also found in association with this class. The **Newly Cultivated Fields **land cover consisted of patches of land recently cultivated. Crops in this class were typically maize under one meter tall, sorghum, and peanuts. Land cover of this type was commonly located adjacent to or between mature maize fields. There were significantly large areas in between the crops where wet, bare soil was exposed but it was classified as newly cultivated fields given the obvious association. Non-cultivated subclasses that were spectrally similar to the newly cultivated fields included areas covered by sedges with surface water under them and fallow lands. Therefore, the vegetative communities that aside from crops were associated with this land cover included *Trichodesma zeylanicum *(Asteraceae), *Cyperus articulatus *(Cyperaceae), *Commelina africana *(Commelinaceae), *Cyperus ajax *(Cyperaceae), *Pennisetum purpuneum *(Napier grass), *Sesbania sesban *(Papillionaceae), *Malva verficillata *(Malvaceae) and Onagraceae spp. Vegetative communities in the **Short Shrubs **land cover were comprised of perennial and herbaceous plant species that were mainly less than 2 meters in height. Some of the dominant plant species included *Sphaeranthus suaveolens *(Asteraceae), *Solanum incanum *(Solanaceae), *Stachytarpheta jamaicensis *(Verbanaceae), *Catharanthus roseus *(Apocynaceae), *Ocimum basilicum, Senna *(*Cassia) occidetalis *(Ceasalpinaceae), *Therettia thevetoides *(Apocenaceae), and *Methania angustifolia *(Sterculaceae). Goats commonly browsed these plants, suggesting an agricultural use. The vegetative communities in the **Tall Shrubs **land cover were dominated by various species of tall shrubs and trees with an average height of over 6 meters. *Justicia flava *(Acanthaceae), *Euphorbia tirucali *(Euphorbiaceae), *Acacia polyacantha *(Mimosaceae) were some of the common plant species associated with this land cover type. In some cases the plants formed a closed canopy. Mango trees (*Mangifera *spp., Anacardiaceae) and banana plants (*Musa *spp., Musaceae), both cultivated for their fruit, occurred in this land cover class. Vegetative communities of the **Medium Shrubs **class were dominated by perennial plants with a mean height of 3–6 meters, especially *Lantana camara *(Verbenaceae), but also *Indigofera spicata *(Papillionaceae), *Ipomoea spathulata *(Convolvulaceae), *Nerium oleander *(Apocynaceae), and *Senna *(*Cassia) floribunda *(Ceasalpinaceae). One obvious use of this land cover type was in border plantings, where oleander and lantana were planted in hedgerows. The majority of streams and still water bodies (such as man-made ponds) were undetectable by the satellite imagery, either because of a closed vegetation canopy (streams) or because the spectral characteristics were similar to bare soil because of turbid water. Therefore, most true bodies of water fell within other land cover classes, and in any case **Open Water ***per se *was an uncommon land cover in the study area and was not included as a classified category. Cloud shadows were found mainly on the northwest area of the image, outside of the study grid. Areas covered by shadow within the study area were primarily as a result of shadows cast by tall trees and small buildings, so the **Shadows **land cover type was incorporated into the tall shrubs or bare soil land covers, respectively.

### Accuracy of image classification

The overall accuracy of the land cover classification from the IKONOS multispectral image was 89% (Kappa statistic, 0.86). The confusion matrix showed that the IKONOS classification was best at distinguishing mature maize and grassland land cover types (Table 1). Generally, the frequency of confusion of classification was low but did occur; for instance, medium shrubs were sometimes confused with both maize and tall shrubs, and turbid water was confused with bare land. Bare land had the lowest classification accuracy because it was largely confused with mature maize, grassland and short shrubs (Table 1). Overall, instances of confusion were minimal and did not affect user's classification accuracy (Table 2). The user's accuracy ranged between 77 and 95%, with relatively low errors of commission (excesses), varying between 5 and 23% (Table 2). The producer's accuracy ranged between 85 and 93%, with similarly relatively low errors of omission (deficits) depending on the class (7–15%).

**Table 1:** Confusion matrix of the IKONOS imagery of supervised classification of land cover types in a rural lowland in western Kenya.

	**Ground truth (Pixels)**						
	
**Land cover types**	Maize	Grassland	Short shrubs	Medium shrubs	Tall shrubs	Bare land	**Total**
Maize	243	6	0	3	1	2	**255**
Grassland	7	163	6	0	3	2	**181**
Short shrubs	7	6	141	1	2	2	**159**
Medium shrubs	9	2	3	154	12	0	**180**
Tall shrubs	6	3	2	7	105	0	**123**
Bare land	4	3	3	1	1	41	**53**

**Total**	**276**	**183**	**155**	**166**	**124**	**47**	**951**

**Table 2:** Producer's and user's accuracy levels of the IKONOS imagery of supervised classification of land cover types in a rural lowland in western Kenya.

**Land cover**	**Producer's accuracy (%)**	**Omission errors (%)**	**User's accuracy (%)**	**Commission errors (%)**
Maize	88	12	95	5
Grassland	89	11	90	10
Short shrubs	91	9	89	11
Medium shrubs	93	7	86	14
Tall shrubs	85	15	85	15

Bare land	87	13	77	23

### Land cover classes and larval habitat location

**Figure 2:**
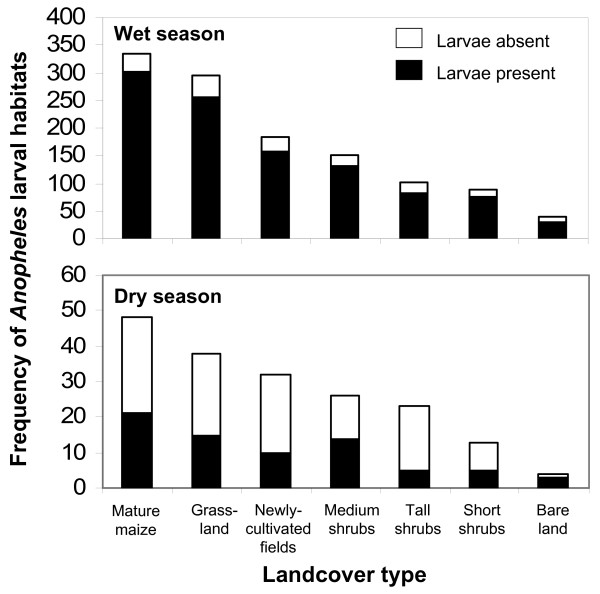
**Frequency of larval *Anopheles *aquatic habitats by land cover type (supervised classification) in a rural landscape in western Kenya, wet season and dry season**. Relative proportion of habitats positive for *Anopheles *larvae is indicated.

Most *Anopheles *larval habitats were located within the mature maize land cover (28%), followed by grasslands (25%), newly cultivated fields (15%), medium shrubs (13%), tall shrubs (9%), short shrubs (7%), and bare land (3%) (Figure 2). There were far more habitats in the wet season compared to the dry season, and more potential habitats were occupied by larvae in the wet season compared to the dry season as well (Figure 2). Assuming a random association, the number of the potential habitats located on each land cover type should be proportional to the total area covered by each land cover type. The expected number of potential habitats by land cover type was calculated within the study area using the method of proportions and using total area of each type as the denominator. It was compared with the observed number of each habitat type with a χ^2 ^test. Overall, there were significant differences between the numbers of the observed and expected potential habitats (χ^2 ^= 324.2, df = 6, P < 0.0001). Approximately 68% of all potential habitats were associated with three of the land cover types, namely mature maize, newly cultivated fields, and grassland. The three land covers had a higher number of the habitats than the expected by chance alone (Table 3). If the habitats had been randomly distributed, 46% of them would be expected to be found in these land cover types. For the remaining four land covers, i.e., short shrubs, medium shrubs, tall shrubs and bare land, there were fewer larval habitats than expected by chance (Table 3).

**Table 3:** Number of observed and expected larval *Anopheles *habitats by land cover type in a rural lowland in western Kenya.

**Land cover**	**Observed habitat counts (%)**	**Expected habitat counts (%)**	**χ^2^**	**P**
Mature Maize	336 (28)	172 (14)	156.37	***
Grassland	294 (25)	255 (21)	5.96	NS
Newly cultivated fields	187 (15)	129 (11)	24.31	**
Short shrubs	90 (7)	174 (15)	40.55	***
Medium shrubs	152 (13)	194 (16)	9.09	NS
Tall shrubs	103 (9)	156 (13)	18.01	**
Bare land	38 (3)	118 (10)	54.24	***

**Total**	**1198 **(100)	**1198 **(100)		

*** indicates differences of chi squared test at significance level of P < 0.001.

** At significance level of P < 0.01.

* At significance level of P < 0.05.

NS-not significant

### Land cover, distance to stream channels, and habitat location

**Figure 3:**
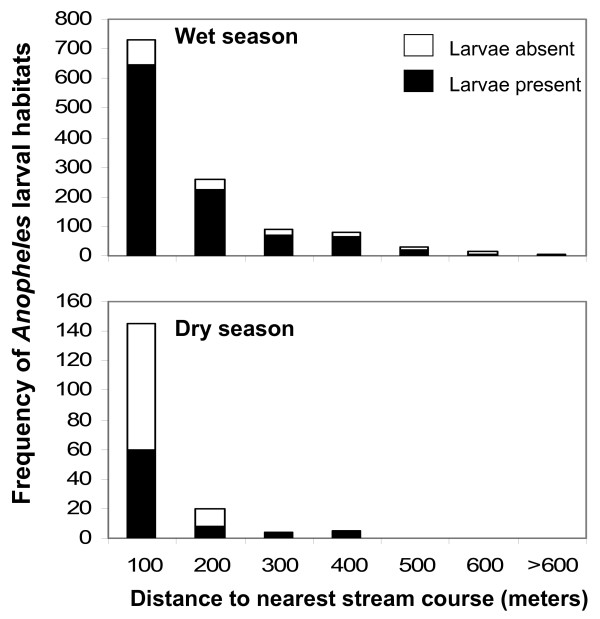
**Frequency of larval *Anopheles *aquatic habitats as a function of distance (meters) from streams in a rural landscape in western Kenya, wet season and dry season**. Relative proportion of habitats positive for *Anopheles *larvae is indicated.

Larval habitat location varied with distance from stream channels in both wet and dry seasons (Figure 3). In the wet season, 82% of all potential habitats and 72% of *Anopheles*-positive larval habitats were located within 200 meters of stream channels. In the dry season, 94% of all potential habitats and 93% of *Anopheles*-positive larval habitats were located within 200 meters of stream channels. Nonlinear regression showed that the number of habitats *y *per 100 m distance from streams *x *fit well the exponential equation: *y *= 1580*e*^-0.0084*x *^(R^2 ^= 0.97). For the wet season, χ^2 ^analysis for categorical variables showed that there were significant differences in the proportions of *Anopheles*-positive larval habitats among the distance categories (χ^2 ^= 25, df = 6, P = 0.0003). χ^2 ^analysis for categorical variables was not done for the dry season data because most of the distance categories had fewer than 5 potential habitats.

**Figure 4:**
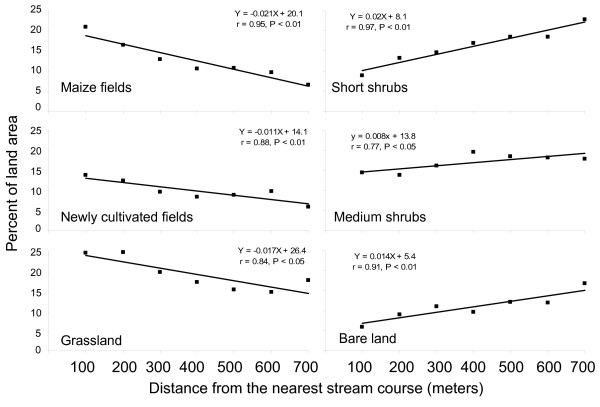
**Percentage of land area occupied by 6 different land cover types (supervised classification) as a function of distance (meters) from streams in a rural landscape in western Kenya**. Tall shrubs land cover not shown (slope from regression not different from 0).

The relationship between the proportion of the various land cover classes and the distance categories from stream channels was examined to determine whether the association of particular land cover types with habitat locations might also be explained by distribution of land cover types relative to streams. Regression analysis showed that area of land covered by grassland, newly cultivated fields, and mature maize were all higher nearer streams and decreased away from streams (Figure 4). By contrast, the area of land covered by bare land, short shrubs, and medium shrubs increased with increasing distance from streams (Figure 4). There was no relationship between area of land covered by tall shrubs and distance from stream channels (r = 0.45, df = 5, P > 0.05).

### Identifying location of water bodies using IKONOS imagery

Within the Asembo study area (area = 10.34 km^2^), supervised classification of the multispectral 4-meter IKONOS image identified water on only 128 pixels. These pixels were extracted from the classified image and converted into polygons using ArcGIS 9.1 (ESRI, Redlands, California), a process that resulted in 27 water bodies (Table 4). To confirm that the IKONOS image truly identified water bodies that were potential larval habitats, a verification procedure was carried out in the field. Maps of the water bodies were prepared and loaded in a personal digital assistant (PDA), which was then used as a navigation guide to each of them. The verification process revealed that 44% (12/27) of the water bodies were rain pools, 30% (8/27) were burrow pits, 22% (6/27) were stream courses, and 1/27 (4%) was a puddle in an automobile tire track.

**Table 4:** Distribution of habitats as identified by ground survey, IKONOS data and by both ground survey and IKONOS in different area categories

**Habitat surface area size (m^2^)**	**Ground survey**	**IKONOS**	**Ground survey + IKONOS**
<1	119	0	0
1 – 3	385	0	0
3 – 10	491	0	0
10 – 20	134	18	10
20 – 40	49	2	0
≥ 40	20	1	1

**Total**	**1,198**	**21**	**11**

## Discussion

Inspection of the results of the supervised classification of land cover types here (Figure 1B) reveals a highly fragmented and anthropogenically-altered landscape with intensive agricultural land use. In nearby Nyando district, on the south side of the Winam Gulf, a similar landscape has experienced substantial edaphic degradation due to intensive agricultural land use with poor soil conservation practices [33,34]. That bare soil forms a major land cover type in our study would suggest similar degradation. Given the consolidation of the unsupervised classification into a relatively simple supervised classification of 7 generalized land cover types here, it is highly unlikely that any major land cover type was missed or that a significant proportion of land cover under study was left unclassified. Indeed, Figure 1B reveals virtually complete coverage with little ambiguity in classification. Further, the botanical characterization reported here, although qualitative, shows that each of the 7 land covers had distinctly dominant plant communities, independently supporting the results of the classification. Generally speaking, the supervised classification process proceeded well in that errors of omission and commission were low and resultant classification accuracy was high. The accuracy of the supervised classification is overestimated because the ground truth points used for the training signatures were also used for verification. However, the proportion correctly classified (or PCC) value was 89% and the Kappa statistic was 0.86, indicating very likely that the classification contained little inflation due to random assignments in the classification process. So, although the accuracy assessment is not optimum, the likelihood that it is significantly worse is low. Further, the supervised classification was supported by an unsupervised classification separately from the ground truth data points, as well as from visual interpretation of the pan-sharpened image. Similar supervised classification studies elsewhere in Kenya are few; a land cover assessment in the River Njoro watershed of the Mau Forest Complex in central Kenya had relatively poorer producer's (50.50%) and user's (66.50%) accuracies compared to our study, perhaps because a 30 m resolution LandSat image was used, but the results were still useful in a land cover change analysis [35].

There were marked influences of land cover type on the location of *Anopheles *larval habitats in the study area. Certain land cover types representing agricultural land uses (including mature maize, grazed grasslands and newly cultivated fields) were positively associated with presence of potential habitats and with *Anopheles *positive habitats, whilst other land covers of mainly non-agricultural uses (including short shrubs, tall shrubs, medium shrubs, and bare land) were negatively associated with the presence of potential habitats. Such a clustered (i.e., overdispersed) distribution of *A. gambiae *larval habitats (see Figure 1A) is often extreme with a high index of aggregation and with spatially-confined pupal production [8,9]. Agricultural lands in the study area represented 46% of the total area but accounted for 68% of the potential habitats and 71% of all larval-positive samples. Potential larval habitats were also located nearer streams, where agricultural land covers were most common, in particular maize, a distribution described well by a convex, monotonically decreasing exponential curve. Maize plantings were likely close to streams with ready access to water for irrigation, were typically flatter and therefore easier to cultivate, and likely had rich alluvial soils amenable to maize plantings. This type of "bottomland" planting is a characteristic of subsistence agriculture in western Kenya, where water flows down slope, eroding soil and forming alluvial deposits [36,37].

The proliferation of *A. gambiae *s.l. larval habitats in the study area was previously shown to be highly associated with human activities, especially agricultural ones [8]. Agricultural activities likely influence availability and suitability of the larval habitats in several ways. First, *A. gambiae *s.l larval habitats elsewhere have been characterized as newly or continuously disturbed, small water bodies [38] such as those created by digging irrigation/drainage channels around cultivated fields [8,9]. Second, larvae of *A. gambiae *have been shown to flourish in these types of larval habitats when they are exposed to direct sun [13,14,39]. Thus, potential habitats located on agricultural lands provided a more suitable environment for breeding because hand dug channels alongside the plantings were present, often held water, and were sun-exposed. The small and shallow water bodies preferred by *An. gambiae *s.l are unshaded and thus receive direct sunlight radiation, which results in optimal temperatures for growth and development of the larvae [12-14] and facilitates proliferation of algae which are an important larval food source [23,40]. Thomas and Lindsay [41] observed that 'pooled sediment' which consisted of exposed beds of alluvial sediment saturated with water, with frequent pooling and supporting sparse vegetation, was an important breeding site for *A. gambiae *larvae in The Gambia, similar to observations we have made here.

The proportion of different land cover classes varied with distance to streams. Higher proportions of land cover classes positively associated with location of potential habitats (agricultural lands) were observed in areas close to the streams, and these decreased with increasing distance from streams (Figure 4). Conversely, higher proportions of land cover classes negatively associated with the location of potential habitats (non-agricultural lands) were observed further away from stream and the proportions decreased towards the streams (Figure 4). This land cover composition structure seemed to be a consequence of combination of land use and topographical factors. The tall and medium shrubs close to streams were likely cleared to give way to mainly maize cultivation. The results of our study in a lowland area were remarkably similar to those in western Kenya highlands of higher altitude and greater relief, where most productive *A. gambiae *larval habitats were located at the valley bottoms near streams on areas predominantly covered by agricultural crops [10,13,14]. Although larvae do not dwell in flowing waters, the association of habitats of streams suggests a hydrological relationship that interacts with land use practices and natural drainage patterns, resulting in pooling of water and establishment of the kinds of productive habitats we observed.

Several studies have shown that larval habitats may not be readily detectable using remote sensing, but that satellite imagery can be used to localize land covers where habitats are likely to occur [1,19,42,43]. The most important malaria vector in our study region, *A. gambiae*, has been observed to breed in small, still and temporary pools of water that rarely are larger than 40 m [8,9,22], ones likely too small to detect using remote sensing. However, land cover associations can be used as a proxy for larval habitats [44]. Earlier applications of remote sensing in malaria control emphasized suitability of very high resolution remote sensing data for mapping vegetation and land cover [15,45]. Zeilhofer and others [46] found savannah scrub and woodlands were more suitable habitats than pastures or cropland for *A. darlingi *in central Brazil. Other studies have associated elevated adult vector densities with specific land cover types [15,47]. The findings of this study and other recent studies that utilized high resolution imagery and land cover analysis confirmed the usefulness of these tools in locating areas with high probabilities of larval habitat location in western Kenya [10,13], Korea [48], Thailand [49], Indonesia [50], and Belize [51].

Whether potential larval habitats of *An. gambiae *could be directly detected using the very high resolution spectral data such as IKONOS or Quickbird was the second goal of this study. The IKONOS satellite image (spatial resolution, 3.2 m) could be used to identify landscape features on the ground as small as 16 m^2 ^was used to determine how effective multispectral data is in directly detecting potential larval habitats. The performance of the IKONOS data in directly detecting the habitats was evaluated through a verification process. The reasons for the verification process included: (1) to confirm whether the water body existed or not, and (2) to determine habitat type and larval presence. The verification procedure indicated that the IKONOS imagery could be used to identify water in those parts of the stream that were more than 4 meters (pixel size for multispectral IKONOS image) wide. The six stream courses (polygons) were not considered as potential larval habitats because water in the streams flows too fast, particularly during the rainy season when this study was done, for *Anopheles *larvae to breed. The IKONOS imagery was therefore only useful in localization of 21 of the 1,198 (1.75%) water bodies that were potential larval habitats. The poor performance of IKONOS imagery in directly detecting i.e. it only detected about 1% of the larval habitats identified by ground surveys, is to a large degree, due to the spatial resolution of the satellite imagery, which failed to delineate any potential larval habitats less than 10 m^2 ^in size. Ground larval habitats survey showed that 83% of the potential habitats were less than 10 m^2 ^in size. Potential larval habitats that were small and had aquatic vegetation and an overhanging canopy were not captured during IKONOS image classification. Also, about 66% of the potential larval habitats located by ground larval habitats survey may have been missed by the IKONOS data because they were turbid and were classified as bare land.

One way to improve the accuracy of IKONOS data in detecting potential larval habitats is by utilizing multi-temporal IKONOS data to detect the potential habitats over time because the target mosquito larval habitats are very transient and some disappear completely in the dry season [9,22]. However, the cost of very high spatial resolution imagery may preclude the use of multi-temporal IKONOS data in detecting potential larval habitats especially in sub-Saharan Africa where vector control resources are scarce. Use of the IKONOS image was limiting in that it was only able to detect directly a few of the habitats larger than 3.2 × 3.2 m (IKONOS spatial resolution). It overlooked the smaller habitats, which are the majority and the most productive [9]. Achee *et al*. [42] also showed that IKONOS data was not sufficient in predicting the specific locations of *A. darlingi *larval habitats within the Sibun River in Belize. Jacob *et al*. [52] used Quickbird (spatial resolution, 2.4 m) to locate large *A. arabiensis *habitats (0.3–1.0 ha) in rice fields of the Mwea irrigation scheme in central Kenya, but did provide information on whether this very high resolution spectral data could be used to locate the typically smaller *A. gambiae *s.l habitats.

Overall, the results of this study exemplify the close associations that exist between *An. gambiae *s.l larval habitats and the agricultural lands in the study area. Agricultural lands offer biologically meaningful associations that are critical for the survival of *A. gambiae*. It was clearly demonstrated that very high resolution satellite images could be utilized in identifying high probability sites for location of potential larval habitats such areas covered by maize fields and pasturelands, thus, an important role of very high resolution satellite images in malaria vector control is established [10,44]. The generality of the associations reported here should be confirmed with similar studies in nearby areas. The associations explored between land cover and larval habitats in this study can best be utilized in vector control programs in the wet season [53]. A single well timed wet season IKONOS satellite image, the only season in which an image was obtained in this study, may suffice in directing control efforts to areas with high probabilities of having potential larval habitats. The wet season is characterized by numerous suitable larval habitats relatively wide spread across the landscape unlike the dry season when the few larval habitats available are known and confined in or along the streams (Mutuku *et al*. in preparation). The link between larval habitats and land cover can be exploited by control programs to gather information on potential larval habitats more efficiently than by field survey and over vast areas.

## Abbreviations

GPS: Global positioning system; GRP: Ground reference points; NCF: Newly cultivated fields; PDA: Personal digital assistant.

## Competing interests

The authors declare that they have no competing interests.

## Authors' contributions

FMM, MNB, JEG, AWH and EDW obtained the satellite image, mapped the study area, and supervised and conducted field research. FMM performed the supervised classification and analyzed data with AWH, JEG, and EDW. FAA conducted the plant community analysis. JMV and JMM provided logistical support.
